# Prevalence of burnout and its associated factors among medical students during COVID-19 pandemic in Indonesia: A cross-sectional study

**DOI:** 10.1371/journal.pone.0285986

**Published:** 2023-06-29

**Authors:** Besut Daryanto, Jemmy Kurniawan, Wiranigitasari, Felicia Hioe, Pradana Nurhadi, Dearisa Surya Yudhantara

**Affiliations:** 1 Department of Urology, Faculty of Medicine, Universitas Brawijaya, Malang City, East Java, Indonesia; 2 Faculty of Medicine, Universitas Brawijaya, Malang City, East Java, Indonesia; 3 Department of Psychiatry, Faculty of Medicine, Universitas Brawijaya, Malang City, East Java, Indonesia; Girne American University - Karmi Campus: Girne Amerikan Universitesi, CYPRUS

## Abstract

Shifting learning process due to COVID-19 has led to increased burnout prevalence among medical students. Thus, this study aimed to assess burnout prevalence and its associated factors among Indonesian medical students during the COVID-19 pandemic. An online cross-sectional study was carried out among medical students in Malang, Indonesia. Burnout was assessed using the Maslach Burnout Inventory-Student Survey tool. Pearson’s Chi-square was used to assess significant associations, and binary logistic regression was conducted to evaluate the relationships between the predictor variables and burnout. The difference of each subscale score was evaluated using an independent sample *t* test. This study analyzed 413 medical students with a mean age of 21.1 ± 1.4 years old. About 29.5% and 32.9% of students reported a high level of emotional exhaustion and depersonalization, respectively, resulting in 17.9% of burnout prevalence. Stage of study was the only sociodemographic factor independently associated with burnout prevalence (odds ratio = 0.180, 95% confidence interval = 0.079–0.410, *p*-values = 0.000). Preclinical students had significantly higher levels of emotional exhaustion (*p*-value = 0.004, d = 0.3) and depersonalization (*p*-value = 0.000, d = 1.1), and lower levels of personal accomplishment (*p*-value = 0.000, d = -0.5). Around one-sixth of the medical students experienced burnout during the COVID-19 pandemic, with preclinical students being more prone to have burnout. Future study with other adjusted confounding factors is needed to completely understand the issue and obtain immediate interventional strategies to reduce burnout among medical students.

## Introduction

Since 2020, Indonesia has been suffering from the coronavirus disease 2019 (COVID-19) pandemic which has an impact on many facets of life, including health, psychological, social, economic, and political issues [1, 2]. Furthermore, the national education system was severely affected as all institutions have been directed to switch the educational process from face-to-face learning to online learning [3]. The new learning process has provided new challenges to the medical students since they need to promptly improve and update their current body of knowledge during the unprecedented times, as well as to ensure the preparation to be qualified medical doctors by possessing all standard competencies despite limited clinical exposures and bedside teaching opportunities [4]. Recently, Mheidly et al demonstrated that online education requiring continuous exposure to a computer or other device has been associated with burnout, elevated stress levels, and stress-related mental and physical symptoms, all of which have an influence on the quality of life and daily activities of the medical students [5].

In recent years, there has been an increase in the number of reports of burnout among medical students [6]. It was firstly described by Kafry and Pines in 1980 as a syndrome characterized by a loss of interest in studying, a lack of motivation, and exhaustion [7]. Medical students were shown to be at a greater risk of burnout than the age-matched individuals of general population, which might be attributable to the stressful nature of medical school, worse logical quality of life, and inadequate coping strategies [8, 9]. A recent systematic review and meta-analysis of medical students from the United States, Australia, and Europe discovered that the prevalence of burnout varied widely, ranging from 7.0% to 75.2% [10]. Unfortunately, it has a detrimental effect on academic achievement as well as professional behavior, which can be aggravated throughout this pandemic era [8]. There are several coping strategies for burnout, but early detection of this syndrome remains the most effective way to prevent and lower its impacts on medical students [11].

Burnout rates among Indonesian residents amid the COVID-19 pandemic have been addressed in previous studies [12, 13]. However, the assessment of burnout among Indonesian medical students during the pandemic era was still unknown. Thus, this study aimed to investigate the prevalence of burnout and its associated factors among medical students in their preclinical and clinical years of study at one of the leading public universities in Indonesia during the COVID-19 pandemic.

## Materials and methods

An online cross-sectional study was conducted on medical students of a state university in Malang, Indonesia, between September 1 and November 1, 2021. The investigation was done using a stratified random sampling technique. The authors distributed the online questionnaires via WhatsApp groups of the respective study years with the explanation about the ethical considerations, data collection, and study procedure. All students were given written informed consent emphasizing that the confidentiality of the identity would be ensured. Ethical approval was obtained from the Health Research Ethics Committee of the Faculty of Medicine, Universitas Brawijaya, after the ethical review conducted in accordance with the Declaration of Helsinki. The inclusion criteria were medical students pursuing Bachelor’s Degree Study Program of Medicine for the 2021/2022 academic year. Other students from other study programs were excluded from the study. Students with duplicate responses were also excluded. Three hundred and fifteen samples were needed as a minimum effective sample size estimated using RaoSoft® (Raosoft, Inc., Seattle, Washington, USA), an online calculator, with a 95% confidence interval (CI), a 5% margin of error, and the prevalence of burnout of 50%.

The questionnaire consisted of questions about identity, survey agreement, sociodemographic characteristics (age, gender, stage of study), and the three categories of burnout symptoms according to the Maslach Burnout Inventory-Student Survey (MBI-SS). Fifteen items measuring emotional exhaustion (EE) subscale (five items), depersonalization (DP) subscale (four items), and personal accomplishment (PA) subscale (six items), were provided on a 7-point Likert scale ranging from 0 to 6 (0 = never, 1 = few times per year, 2 = once a month, 3 = few times per month, 4 = once a week, 5 = few times per week, 6 = everyday). Low, moderate, and high levels of each subscale were identified using an arbitrary statistical cut-off criterion based on percentile 33^rd^ and percentile 66^th^, respectively. Burnout in this study was identified from high levels of both EE and DP as specified in the eleventh revision of the International Classification of Diseases for the two-dimensional model. The internal consistency of the MBI-SS tool was obtained using Cronbach’s alpha.

Data analyses were conducted using the IBM SPSS version 26 (IBM SPSS Statistics for Windows, IBM Corporation, Armonk, NY). The categorical variables of the medical students were reported as numbers and percentages, while continuous data were presented in mean ± standard deviation (SD). The significant associations between the frequency of categorical variables were analyzed using Pearson’s Chi-square. The binary logistic regression was further used to evaluate the predictive relationship between burnout and the associated factors. Adjusted odds ratio (OR) and 95% CI were also assessed. Independent sample *t* test was performed to compare the mean difference of each MBI-SS subscale score between the categorical independent variables. Welch test was reported when the homogeneity of variances assumption of Levene’s test was not met for each variable. Effect size was measured using Cohen’s proposal, which interpreted d = 0.2, d = 0.5, and d = 0.8 as small, medium, and large, respectively [14]. A *p*-value <0.05 was considered significant.

## Results

Four hundred and twenty-five medical students responded. Twelve duplicate responses were removed, leaving 413 (24.0% response rate) medical students. Of whom, 274 medical students were in their preclinical years, while the remaining 139 were in their clinical years. More than three-fifths (63.0%) of the students were aged 21 and over with a mean age of 21.1 ± 1.4 years old. The majority of the students were female (70.9%). Further information about the characteristics of the study participants can be seen in Table 1.

**Table 1:** Sociodemographic of the medical students.

Characteristics		n (%)
**Total number of participants**		413 (100.0)
**Age in years**	Mean ± standard deviation	21.1 ± 1.4
	<21	153 (37.0)
	≥21	260 (63.0)
**Gender**	Male	120 (29.1)
	Female	293 (70.9)
**Stage of study**	Preclinical	274 (66.3)
	Clinical	139 (33.7)

The majority of the students stated a moderate level of EE (10–17), DP (5–11), and PA (17–23) with the mean subscale score of 14.0 ± 6.6, 8.5 ± 6.3, and 20.4 ± 6.6, respectively. About 29.5% and 32.9% of students reported a high level of EE (>17) and DP (>11), respectively, resulting in 17.9% of the burnout prevalence among the students. Additionally, the reliability test (Cronbach’s alpha) for the three subscales of the MBI-SS of the study was greater than 0.700 (EE = 0.927, DP = 0.937, and PA = 0.862), indicating adequate internal consistency (Table 2).

**Table 2:** Distribution of MBI–SS and the prevalence of burnout.

Indicators			n (%)
**MBI-SS** ^**†**^	EE	Mean ± standard deviation	14.0 ± 6.6
		Low (<10)	102 (24.7)
		Moderate (10–17)	189 (45.8)
		High (>17)	122 (29.5)
	DP	Mean ± standard deviation	8.5 ± 6.3
		Low (<5)	134 (32.4)
		Moderate (5–11)	143 (34.6)
		High (>11)	136 (32.9)
	PA	Mean ± standard deviation	20.4 ± 6.6
		Low (>23)	134 (32.4)
		Moderate (17–23)	173 (41.9)
		High (<17)	106 (25.7)
**Burnout**	Yes		74 (17.9)
	No		339 (82.1)

^**†**^Cronbach’s alpha for EE, DP, and PA of MBI-SS were 0.927, 0.937, and 0.862, respectively. MBI-SS = Maslach Burnout Inventory-Student Survey, EE = emotional exhaustion, DP = depersonalization, PA = personal accomplishment.

**Fig 1:**
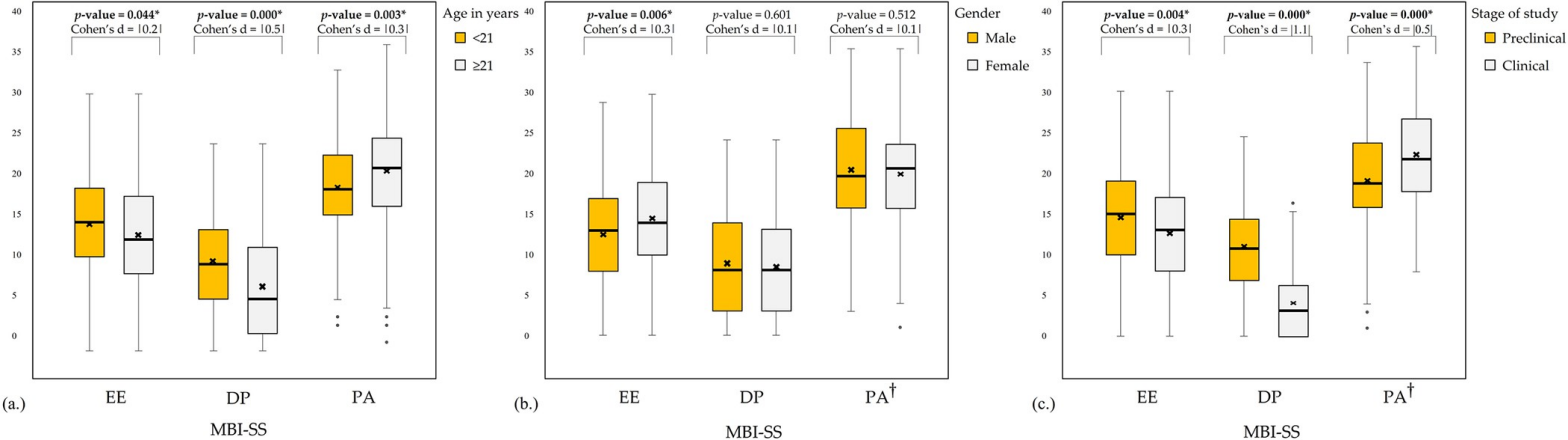
Mean differences between age groups (a), gender (b), and stage of study (c) on MBI-SS subscale scores. †Welch test is reported because Levene’s test indicated that the homogeneity of variances assumption was not met for this variable. *Significant at p-value <0.05. MBI-SS = Maslach Burnout Inventory-Student Survey, EE = emotional exhaustion, DP = depersonalization, PA = personal accomplishment.

Fig 1(A) demonstrated the comparison of mean differences according to the age groups for each MBI-SS subscale score. Medical students who were under the age of 21 years had significantly higher levels of both EE (14.8 ± 6.5 versus 13.5 ± 6.6, *p*-value = 0.044, d = 0.2) and DP (10.4 ± 5.8 versus 7.4 ± 6.4, *p*-value = 0.000, d = 0.5) compared to the older group. The mean score for PA levels, on the other hand, was found to be significantly lower in the younger group compared to the older group (19.2 ± 6.3 versus 21.2 ± 6.6, *p*-value = 0.003, d = -0.3).

The level of EE in male students was significantly lower than female students (12.6 ± 6.6 versus 14.5 ± 6.5, *p*-value = 0.006, d = -0.3). However, the different level of both DP (8.8 ± 6.6 versus 8.4 ± 6.2, *p*-value = 0.601, d = 0.1) and PA (20.8 ± 7.3 versus 20.3 ± 6.2, *p*-value = 0.512, d = 0.1) between male and female students were not statistically significant. Moreover, the effect size (Cohen’s d) for both DP and PA between genders was less than 0.2, indicating that the difference between these groups was negligible (Fig 1(B)).

Fig 1(C) showed the mean differences on MBI-SS subscale scores between preclinical and clinical years. Both EE and DP levels were higher among the preclinical medical students compared to the clinical medical students (14.6 ± 6.6 versus 12.7 ± 6.3, *p*-value = 0.004, d = 0.3 and 10.8 ± 6.0 versus 4.0 ± 4.3, *p*-value = 0.000, d = 1.1, respectively). Meanwhile, the preclinical medical students had significantly lower levels of PA compared to the clinical medical students (19.3 ± 6.4 versus 22.6 ± 6.4, *p*-value = 0.000, d = -0.5).

The association between medical students’ sociodemographic factors and burnout as well as the logistic regression analysis of burnout prevalence were shown in Table 3. The tabulation demonstrated that both age and stage of study were associated with the prevalence of burnout (*p*-value = 0.023 and *p*-value = 0.000, respectively). There was no association between burnout prevalence and gender (*p*-value = 0.120). After performing the logistic regression analysis, the only sociodemographic factor independently associated with burnout prevalence was stage of study (OR = 0.180, 95% CI = 0.079–0.410, *p*-values = 0.000).

**Table 3:** Association between the sociodemographic of the medical students and burnout prevalence with the logistic regression analysis.

Sociodemographic factors		Burnout		*p*-value	Logistic regression analysis		
		Yes	No		OR	95% CI	*p*-value
**Age in years**	<21	36	117	0.023*	1.111	0.634–1.948	0.713
	≥21	38	222				
**Gender**	Male	16	104	0.120			
	Female	58	235				
**Stage of study**	Preclinical	66	208	0.000*	0.180	0.079–0.410	0.000*
	Clinical	8	131				

*Significant at *p*-value <0.05. OR = odds ratio, CI = confidence interval

## Discussion

This study established that during the COVID-19 pandemic, 74 (17.9%) medical students suffered from burnout, which is similar to previous study conducted by Zis et al, Adham et al, and Alqifari et al, where the prevalence of burnout among medical students during the COVID-19 era was 18.2%, 15.0%, and 18.0%, respectively [15–17]. However, based on a study conducted by Shrestha et al, using the English version of the Oldenburg Burnout Inventory adapted for students (OLBI-S), the prevalence of burnout among medical students in Kathmandu amid the pandemic was reported as high as 65.9% [18]. This was in conjunction with the findings from a study by Forycka et al reporting that the prevalence of burnout among Polish medical students during the COVID-19 pandemic was around 59.9% [19]. A recent meta-analysis, including all observational studies in China from inception to 2019, exhibited that the prevalence of burnout among medical students was 45.9% (95% CI = 38.1%-53.8%) [20]. This gap between the prevalence might be caused by several factors, such as different criteria for defining burnout, different measurement instruments, timing of the survey, the sociodemographic factors of the study participants, and various effects of ongoing pandemic for each individual and institutional learning system [21, 22].

Evaluating each burnout subscale separately, the prevalence of high EE (>17), DP (>11), and PA (<17) in this study were nearly equal to that of Alqifari et al (EE = 29.5%, DP = 33.3%, PA = 33.9%) [17]. Meanwhile, other studies showed greater prevalence of each subscale score throughout the COVID-19 era [23, 24]. A study by Li et al showed that the prevalence of high EE, DP, and PA were 37.5%, 36.0%, and 44.0%, respectively, prior to the outbreak of COVID-19, which were greater than the prevalence of high burnout subscales of this study [20]. Moreover, Altannir et al reported that the prevalence of high DP and PA in the pre-pandemic era were higher than that of this study, indicating that high DP and PA were found to be lower amid the COVID-19 pandemic, excluding high EE subscales which were found to be higher [25]. In contrast, a study from Cyprus showed that the average DP subscale score increased significantly when compared to pre-pandemic condition and there was no statistically significant difference in EE and PA scores between the two periods [15]. The diversity of the prevalence might also be ascribed to the sociocultural factors that influence each burnout subscale in multiple settings [9, 26]. More studies are needed to determine the underlying reasons for the extensive range of burnout subscales prevalence among medical students.

The result of the binary logistic regression analysis revealed that clinical medical students had an 82% lower likelihood of suffering from burnout during the COVID-19 pandemic compared to the preclinical medical students. This finding contradicted the findings of Kajjimu et al, who concluded that there was no association between stage of study and the prevalence of burnout during the pandemic [24]. In comparison to the pre-pandemic era, two previous studies confirmed that clinical medical students had a higher likelihood of burnout than preclinical medical students [23, 27]. Meanwhile, Altannir et al reported the opposite results, which were consistent with the findings of this study [25]. Zis et al revealed that this shifting condition was influenced by a change in learning methods from the previously rigorous workload of clinical rotations in the hospitals to the distance electronic learning in clinical medical education during the COVID-19 pandemic [15]. Furthermore, the decreased prevalence of burnout among clinical medical students might be attributed to the fact that they had learned to use adaptive coping strategies and had better peer discussion groups [23].

In terms of burnout subscale score, this study showed that as the school year progressed, the mean levels of EE and DP declined while the mean level of PA rose. This was in contrast to the finding of Alqifari et al who discovered that EE level was much greater in the final years of medical students [17]. However, the findings of this study correlated with previous studies undertaken by Altannir et al before the era of COVID-19, in which EE and DP levels fell with study year [25]. In addition, Cecil et al demonstrated a similar outcome in regard to PA subscale score, which was found to be higher among clinical medical students during the pre-pandemic era [28]. As stated by Ramadianto et al., this phenomenon might be attributed to a lower anxiety score that was statistically significant among Indonesian clinical medical students compared to preclinical medical students during the pandemic period [29]. Additionally, as previously reported, the stress levels of Indonesian medical students peak in their first year of education and then steadily subside over the following years, which could contribute to the fact that the mean levels of EE and DP were significantly lower and the mean level of PA was significantly higher in clinical medical students than preclinical medical students in this study [30].

Although age was significantly associated with the prevalence of burnout, this variable was not a predictor of burnout in this study. These findings were supported with other studies undergone during the pandemic which established that age was not independently associated with the prevalence of burnout [19, 23]. However, there were statistically significant mean differences in burnout subscale scores between age groups in this study which was consistent with a study done among Brazilian medical students, which revealed that high EE and DP with lower PA scores were more prevalent in the younger age group [31]. A recent meta-analysis by Frajerman et al stated that young age was postulated as a factor contributing to the higher burnout subscale scores among medical students [27]. Interestingly, several other studies conducted amid the COVID-19 pandemic found no significant differences between age groups regarding burnout subscale scores [17, 23, 24].

Regarding the influence of gender on the prevalence of burnout, this study showed that no association was identified and there were no significant differences in DP and PA scores. However, female gender had significantly higher mean score of EE which was similar to prior studies conducted by Alqifari et al and Aljadani et al who claimed that female gender was a significant predictor of high EE in medical students amid the pandemic era [17, 23]. This might be due to female medical students were more likely to experience stress than male medical students because they face more demands outside of the medical school [32]. Moreover, Mirza et al mentioned that depression and anxiety were more common in females than males, which contributed to the greater burnout subscale scores, particularly for EE [33].

This study introduced the prevalence of burnout among medical students of a state university and its association with age, gender, and stage of study during this unprecedented pandemic. The findings are useful for medical educators in proposing strategies to alleviate burnout, particularly among preclinical medical students, who were shown to have a higher prevalence of burnout in this study. Adaptive education strategies have been widely utilized, however, the benefits and drawbacks of permanently adopting such novel educational approaches should be sufficiently weighted, especially in medical education, which is not suitable for online learning as students must be exposed in a real clinical setting. Employing practical and successful coping methods or other efforts must be taken in this crucial time, since medical students who graduate with burnout are more likely to acquire severe burnout or other mental health disorders throughout their future life as a general practitioner or residency training.

There were some limitations to this study. First, this was a cross-sectional study which did not investigate the prevalence of burnout among medical students prior to the COVID-19 pandemic era. The different workloads of medical students during the survey could also affect the outcomes of this study. Furthermore, the study was performed at a single institution only, that might lead to its limited generalizability. In addition, the response rate of this study was relatively low, which, as with all questionnaire studies, could be caused by the non-response bias. However, as the study population is quite large, a minimum of 18.0% response rate is sufficient for the survey to be representative. Finally, this study did not adjust other potential confounding factors, such as interpersonal stress, personality traits, other psychiatric disorders, excessive fear of COVID-19, family and sociocultural backgrounds, which could cause the variety of outcomes when compared to other studies.

Approximately one-sixth of medical students in this study had burnout during the COVID-19 pandemic. Preclinical medical students were found to be more likely to experience burnout. Future study with adjusted potential confounding factors is needed to fully understand this issue and to devise an effective strategy to minimize the rates of burnout among medical students.

## Supporting information

S1 Dataset (PDF)
