# Supplementary material for: Prevalence of burnout and its associated factors among medical students during COVID-19 pandemic in Indonesia: A cross-sectional study
Source: PLoS One. 2023 Jun 29;18(6):e0285986. doi: 10.1371/journal.pone.0285986 (PMC10309627; doi:10.1371/journal.pone.0285986)
Supplement: S1 Dataset — (PDF) [file pone.0285986.s001.pdf]

Number	Age in years	Gender	Stage of study	EE	Level of EE	DP	Level of DP	PA	Level of PA	Burnout
1	20	Female	Preclinical	16	Moderate	13	High	16	High	Not Burnout
2	20	Male	Preclinical	19	High	16	High	16	High	Burnout
3	20	Male	Preclinical	15	Moderate	15	High	18	Moderate	Not Burnout
4	20	Female	Preclinical	20	High	11	Moderate	24	Low	Not Burnout
5	20	Female	Preclinical	28	High	21	High	13	High	Burnout
6	20	Female	Preclinical	20	High	19	High	14	High	Burnout
7	18	Male	Preclinical	17	Moderate	12	High	18	Moderate	Not Burnout
8	20	Female	Preclinical	18	High	8	Moderate	17	Moderate	Not Burnout
9	20	Female	Preclinical	20	High	12	High	18	Moderate	Burnout
10	21	Female	Preclinical	20	High	12	High	16	High	Burnout
11	21	Female	Preclinical	6	Low	4	Low	29	Low	Not Burnout
12	20	Female	Preclinical	13	Moderate	10	Moderate	23	Moderate	Not Burnout
13	20	Female	Preclinical	7	Low	0	Low	21	Moderate	Not Burnout
14	21	Female	Preclinical	14	Moderate	8	Moderate	20	Moderate	Not Burnout
15	20	Female	Preclinical	14	Moderate	13	High	26	Low	Not Burnout
16	19	Female	Preclinical	11	Moderate	6	Moderate	21	Moderate	Not Burnout
17	20	Female	Preclinical	21	High	14	High	24	Low	Burnout
18	20	Male	Preclinical	16	Moderate	8	Moderate	25	Low	Not Burnout
19	20	Female	Preclinical	10	Moderate	6	Moderate	28	Low	Not Burnout
20	20	Female	Preclinical	14	Moderate	14	High	15	High	Not Burnout
21	20	Female	Preclinical	2	Low	2	Low	16	High	Not Burnout
22	20	Female	Preclinical	12	Moderate	9	Moderate	15	High	Not Burnout
23	20	Male	Preclinical	12	Moderate	6	Moderate	11	High	Not Burnout
24	20	Female	Preclinical	16	Moderate	6	Moderate	20	Moderate	Not Burnout
25	19	Female	Preclinical	18	High	8	Moderate	30	Low	Not Burnout
26	20	Female	Preclinical	18	High	10	Moderate	15	High	Not Burnout
27	19	Female	Preclinical	15	Moderate	10	Moderate	29	Low	Not Burnout
28	19	Male	Preclinical	19	High	11	Moderate	17	Moderate	Not Burnout
29	20	Female	Preclinical	19	High	8	Moderate	20	Moderate	Not Burnout
30	21	Female	Preclinical	30	High	23	High	9	High	Burnout
31	20	Female	Preclinical	16	Moderate	4	Low	23	Moderate	Not Burnout
32	20	Female	Preclinical	4	Low	1	Low	9	High	Not Burnout
33	21	Female	Preclinical	4	Low	0	Low	27	Low	Not Burnout
34	20	Female	Preclinical	13	Moderate	9	Moderate	32	Low	Not Burnout
35	19	Female	Preclinical	9	Low	6	Moderate	12	High	Not Burnout
36	20	Female	Preclinical	16	Moderate	12	High	14	High	Not Burnout
37	20	Male	Preclinical	14	Moderate	12	High	15	High	Not Burnout
38	18	Female	Preclinical	16	Moderate	14	High	18	Moderate	Not Burnout
39	20	Female	Preclinical	8	Low	19	High	20	Moderate	Not Burnout
40	21	Female	Preclinical	30	High	22	High	8	High	Burnout
41	21	Female	Preclinical	7	Low	2	Low	32	Low	Not Burnout
42	21	Female	Preclinical	25	High	13	High	20	Moderate	Burnout
43	20	Female	Preclinical	12	Moderate	20	High	10	High	Not Burnout

44	20	Female	Preclinical	5	Low	6	Moderate	24	Low	Not Burnout
45	20	Male	Preclinical	9	Low	9	Moderate	26	Low	Not Burnout
46	20	Female	Preclinical	17	Moderate	13	High	23	Moderate	Not Burnout
47	19	Female	Preclinical	15	Moderate	14	High	17	Moderate	Not Burnout
48	20	Male	Preclinical	15	Moderate	8	Moderate	19	Moderate	Not Burnout
49	20	Female	Preclinical	8	Low	4	Low	11	High	Not Burnout
50	19	Female	Preclinical	8	Low	5	Moderate	22	Moderate	Not Burnout
51	19	Female	Preclinical	14	Moderate	11	Moderate	22	Moderate	Not Burnout
52	20	Female	Preclinical	13	Moderate	8	Moderate	15	High	Not Burnout
53	20	Female	Preclinical	17	Moderate	0	Low	24	Low	Not Burnout
54	20	Male	Preclinical	6	Low	4	Low	33	Low	Not Burnout
55	20	Female	Preclinical	6	Low	2	Low	23	Moderate	Not Burnout
56	20	Female	Preclinical	20	High	13	High	17	Moderate	Burnout
57	18	Male	Preclinical	15	Moderate	10	Moderate	24	Low	Not Burnout
58	20	Male	Preclinical	16	Moderate	18	High	22	Moderate	Not Burnout
59	20	Female	Preclinical	23	High	11	Moderate	19	Moderate	Not Burnout
60	20	Female	Preclinical	13	Moderate	11	Moderate	19	Moderate	Not Burnout
61	21	Female	Preclinical	14	Moderate	6	Moderate	19	Moderate	Not Burnout
62	20	Female	Preclinical	5	Low	1	Low	22	Moderate	Not Burnout
63	20	Female	Preclinical	12	Moderate	9	Moderate	20	Moderate	Not Burnout
64	20	Female	Preclinical	17	Moderate	12	High	20	Moderate	Not Burnout
65	21	Male	Preclinical	10	Moderate	6	Moderate	27	Low	Not Burnout
66	20	Female	Preclinical	6	Low	5	Moderate	9	High	Not Burnout
67	20	Female	Preclinical	5	Low	4	Low	6	High	Not Burnout
68	20	Female	Preclinical	6	Low	6	Moderate	23	Moderate	Not Burnout
69	20	Female	Preclinical	23	High	9	Moderate	14	High	Not Burnout
70	19	Male	Preclinical	15	Moderate	15	High	15	High	Not Burnout
71	20	Female	Preclinical	1	Low	5	Moderate	21	Moderate	Not Burnout
72	21	Male	Preclinical	18	High	8	Moderate	28	Low	Not Burnout
73	19	Male	Preclinical	29	High	24	High	21	Moderate	Burnout
74	20	Female	Preclinical	13	Moderate	7	Moderate	22	Moderate	Not Burnout
75	20	Male	Preclinical	25	High	20	High	3	High	Burnout
76	20	Female	Preclinical	10	Moderate	3	Low	24	Low	Not Burnout
77	20	Female	Preclinical	23	High	14	High	20	Moderate	Burnout
78	19	Female	Preclinical	23	High	18	High	33	Low	Burnout
79	20	Female	Preclinical	12	Moderate	8	Moderate	24	Low	Not Burnout
80	20	Male	Preclinical	20	High	20	High	16	High	Burnout
81	19	Male	Preclinical	13	Moderate	14	High	16	High	Not Burnout
82	19	Male	Preclinical	6	Low	7	Moderate	17	Moderate	Not Burnout
83	20	Female	Preclinical	19	High	12	High	18	Moderate	Burnout
84	20	Female	Preclinical	18	High	15	High	10	High	Burnout
85	20	Female	Preclinical	23	High	12	High	21	Moderate	Burnout
86	21	Female	Preclinical	13	Moderate	7	Moderate	21	Moderate	Not Burnout
87	20	Female	Preclinical	23	High	24	High	19	Moderate	Burnout
88	20	Female	Preclinical	25	High	21	High	15	High	Burnout
89	20	Female	Preclinical	25	High	19	High	23	Moderate	Burnout
90	20	Female	Preclinical	20	High	15	High	25	Low	Burnout

91	19	Male	Preclinical	12	Moderate	10	Moderate	17	Moderate	Not Burnout
92	20	Female	Preclinical	11	Moderate	6	Moderate	25	Low	Not Burnout
93	20	Female	Preclinical	18	High	13	High	17	Moderate	Burnout
94	19	Female	Preclinical	17	Moderate	10	Moderate	17	Moderate	Not Burnout
95	20	Female	Preclinical	17	Moderate	14	High	14	High	Not Burnout
96	20	Female	Preclinical	24	High	12	High	19	Moderate	Burnout
97	20	Female	Preclinical	19	High	10	Moderate	15	High	Not Burnout
98	20	Male	Preclinical	9	Low	16	High	4	High	Not Burnout
99	20	Female	Preclinical	14	Moderate	4	Low	23	Moderate	Not Burnout
100	20	Female	Preclinical	11	Moderate	8	Moderate	23	Moderate	Not Burnout
101	22	Female	Preclinical	12	Moderate	14	High	19	Moderate	Not Burnout
102	20	Male	Preclinical	0	Low	6	Moderate	31	Low	Not Burnout
103	20	Female	Preclinical	28	High	7	Moderate	24	Low	Not Burnout
104	20	Female	Preclinical	9	Low	4	Low	20	Moderate	Not Burnout
105	19	Female	Preclinical	6	Low	6	Moderate	30	Low	Not Burnout
106	19	Female	Preclinical	30	High	23	High	10	High	Burnout
107	20	Female	Preclinical	5	Low	4	Low	24	Low	Not Burnout
108	20	Female	Preclinical	25	High	23	High	14	High	Burnout
109	19	Male	Preclinical	10	Moderate	14	High	16	High	Not Burnout
110	20	Male	Preclinical	5	Low	4	Low	33	Low	Not Burnout
111	19	Female	Preclinical	17	Moderate	12	High	10	High	Not Burnout
112	20	Male	Preclinical	14	Moderate	9	Moderate	21	Moderate	Not Burnout
113	20	Female	Preclinical	23	High	21	High	7	High	Burnout
114	20	Female	Preclinical	21	High	4	Low	29	Low	Not Burnout
115	20	Male	Preclinical	23	High	8	Moderate	26	Low	Not Burnout
116	20	Female	Preclinical	2	Low	0	Low	31	Low	Not Burnout
117	20	Female	Preclinical	20	High	17	High	17	Moderate	Burnout
118	21	Male	Preclinical	21	High	9	Moderate	22	Moderate	Not Burnout
119	19	Female	Preclinical	9	Low	12	High	23	Moderate	Not Burnout
120	20	Female	Preclinical	14	Moderate	8	Moderate	22	Moderate	Not Burnout
121	20	Female	Preclinical	17	Moderate	9	Moderate	20	Moderate	Not Burnout
122	20	Female	Preclinical	15	Moderate	13	High	22	Moderate	Not Burnout
123	21	Female	Preclinical	12	Moderate	14	High	26	Low	Not Burnout
124	20	Female	Preclinical	8	Low	6	Moderate	6	High	Not Burnout
125	20	Female	Preclinical	18	High	13	High	15	High	Burnout
126	20	Female	Preclinical	12	Moderate	4	Low	28	Low	Not Burnout
127	20	Male	Preclinical	9	Low	7	Moderate	30	Low	Not Burnout
128	20	Male	Preclinical	13	Moderate	12	High	11	High	Not Burnout
129	19	Female	Preclinical	15	Moderate	10	Moderate	19	Moderate	Not Burnout
130	20	Male	Preclinical	23	High	20	High	18	Moderate	Burnout
131	20	Female	Preclinical	14	Moderate	24	High	4	High	Not Burnout
132	20	Female	Preclinical	25	High	20	High	18	Moderate	Burnout
133	20	Male	Preclinical	5	Low	0	Low	25	Low	Not Burnout
134	20	Female	Preclinical	12	Moderate	5	Moderate	18	Moderate	Not Burnout
135	20	Male	Preclinical	22	High	15	High	21	Moderate	Burnout
136	20	Male	Preclinical	15	Moderate	13	High	19	Moderate	Not Burnout
137	21	Female	Preclinical	15	Moderate	12	High	16	High	Not Burnout

138	19	Female	Preclinical	21	High	14	High	17	Moderate	Burnout
139	21	Female	Preclinical	23	High	19	High	23	Moderate	Burnout
140	21	Male	Preclinical	16	Moderate	22	High	11	High	Not Burnout
141	21	Female	Preclinical	20	High	10	Moderate	28	Low	Not Burnout
142	21	Female	Preclinical	17	Moderate	10	Moderate	22	Moderate	Not Burnout
143	22	Male	Preclinical	24	High	18	High	8	High	Burnout
144	22	Male	Preclinical	16	Moderate	12	High	18	Moderate	Not Burnout
145	21	Male	Preclinical	9	Low	1	Low	34	Low	Not Burnout
146	21	Male	Preclinical	11	Moderate	17	High	17	Moderate	Not Burnout
147	21	Male	Preclinical	8	Low	8	Moderate	23	Moderate	Not Burnout
148	21	Female	Preclinical	16	Moderate	10	Moderate	26	Low	Not Burnout
149	22	Female	Preclinical	13	Moderate	11	Moderate	16	High	Not Burnout
150	21	Female	Preclinical	27	High	2	Low	32	Low	Not Burnout
151	17	Female	Preclinical	10	Moderate	3	Low	24	Low	Not Burnout
152	21	Male	Preclinical	10	Moderate	13	High	17	Moderate	Not Burnout
153	21	Female	Preclinical	30	High	20	High	4	High	Burnout
154	21	Female	Preclinical	9	Low	10	Moderate	24	Low	Not Burnout
155	21	Male	Preclinical	24	High	20	High	5	High	Burnout
156	21	Male	Preclinical	11	Moderate	1	Low	26	Low	Not Burnout
157	21	Female	Preclinical	13	Moderate	0	Low	24	Low	Not Burnout
158	21	Female	Preclinical	15	Moderate	6	Moderate	21	Moderate	Not Burnout
159	21	Female	Preclinical	12	Moderate	8	Moderate	15	High	Not Burnout
160	20	Female	Preclinical	12	Moderate	0	Low	26	Low	Not Burnout
161	21	Female	Preclinical	21	High	19	High	20	Moderate	Burnout
162	22	Male	Preclinical	11	Moderate	3	Low	18	Moderate	Not Burnout
163	21	Female	Preclinical	8	Low	0	Low	32	Low	Not Burnout
164	21	Female	Preclinical	12	Moderate	16	High	20	Moderate	Not Burnout
165	21	Female	Preclinical	4	Low	2	Low	29	Low	Not Burnout
166	19	Female	Preclinical	21	High	13	High	20	Moderate	Burnout
167	21	Female	Preclinical	19	High	15	High	15	High	Burnout
168	20	Male	Preclinical	19	High	8	Moderate	24	Low	Not Burnout
169	21	Female	Preclinical	9	Low	11	Moderate	19	Moderate	Not Burnout
170	21	Female	Preclinical	16	Moderate	7	Moderate	22	Moderate	Not Burnout
171	20	Female	Preclinical	18	High	18	High	6	High	Burnout
172	22	Female	Preclinical	23	High	10	Moderate	14	High	Not Burnout
173	21	Female	Preclinical	15	Moderate	19	High	19	Moderate	Not Burnout
174	22	Female	Preclinical	17	Moderate	12	High	19	Moderate	Not Burnout
175	21	Female	Preclinical	20	High	20	High	14	High	Burnout
176	21	Female	Preclinical	11	Moderate	10	Moderate	29	Low	Not Burnout
177	21	Female	Preclinical	7	Low	10	Moderate	13	High	Not Burnout
178	21	Male	Preclinical	16	Moderate	13	High	18	Moderate	Not Burnout
179	20	Female	Preclinical	13	Moderate	11	Moderate	16	High	Not Burnout
180	21	Male	Preclinical	27	High	24	High	9	High	Burnout
181	20	Male	Preclinical	3	Low	0	Low	18	Moderate	Not Burnout
182	20	Female	Preclinical	22	High	15	High	16	High	Burnout
183	21	Female	Preclinical	25	High	15	High	10	High	Burnout
184	21	Female	Preclinical	15	Moderate	13	High	22	Moderate	Not Burnout

185	21	Male	Preclinical	9	Low	9	Moderate	9	High	Not Burnout
186	21	Male	Preclinical	12	Moderate	8	Moderate	19	Moderate	Not Burnout
187	20	Male	Preclinical	13	Moderate	8	Moderate	9	High	Not Burnout
188	21	Female	Preclinical	3	Low	8	Moderate	24	Low	Not Burnout
189	21	Male	Preclinical	11	Moderate	12	High	17	Moderate	Not Burnout
190	21	Female	Preclinical	16	Moderate	0	Low	24	Low	Not Burnout
191	21	Male	Preclinical	19	High	16	High	24	Low	Burnout
192	22	Female	Preclinical	17	Moderate	12	High	18	Moderate	Not Burnout
193	21	Male	Preclinical	19	High	14	High	17	Moderate	Burnout
194	21	Female	Preclinical	19	High	9	Moderate	17	Moderate	Not Burnout
195	21	Female	Preclinical	6	Low	2	Low	20	Moderate	Not Burnout
196	19	Female	Preclinical	17	Moderate	5	Moderate	25	Low	Not Burnout
197	21	Male	Preclinical	19	High	15	High	20	Moderate	Burnout
198	19	Female	Preclinical	12	Moderate	12	High	18	Moderate	Not Burnout
199	22	Male	Preclinical	17	Moderate	7	Moderate	21	Moderate	Not Burnout
200	21	Female	Preclinical	18	High	6	Moderate	17	Moderate	Not Burnout
201	21	Male	Preclinical	2	Low	1	Low	24	Low	Not Burnout
202	21	Male	Preclinical	17	Moderate	9	Moderate	15	High	Not Burnout
203	22	Male	Preclinical	2	Low	9	Moderate	11	High	Not Burnout
204	20	Female	Preclinical	17	Moderate	9	Moderate	23	Moderate	Not Burnout
205	21	Female	Preclinical	26	High	22	High	17	Moderate	Burnout
206	24	Male	Preclinical	3	Low	1	Low	32	Low	Not Burnout
207	23	Male	Preclinical	7	Low	9	Moderate	25	Low	Not Burnout
208	21	Female	Preclinical	13	Moderate	0	Low	24	Low	Not Burnout
209	20	Female	Preclinical	0	Low	15	High	16	High	Not Burnout
210	21	Male	Preclinical	17	Moderate	23	High	3	High	Not Burnout
211	21	Female	Preclinical	19	High	19	High	13	High	Burnout
212	21	Female	Preclinical	17	Moderate	6	Moderate	27	Low	Not Burnout
213	21	Female	Preclinical	6	Low	14	High	17	Moderate	Not Burnout
214	21	Male	Preclinical	5	Low	21	High	25	Low	Not Burnout
215	19	Female	Preclinical	15	Moderate	11	Moderate	20	Moderate	Not Burnout
216	21	Male	Preclinical	3	Low	12	High	15	High	Not Burnout
217	22	Male	Preclinical	12	Moderate	8	Moderate	12	High	Not Burnout
218	20	Female	Preclinical	0	Low	0	Low	30	Low	Not Burnout
219	22	Female	Preclinical	15	Moderate	13	High	17	Moderate	Not Burnout
220	21	Female	Preclinical	18	High	6	Moderate	22	Moderate	Not Burnout
221	21	Female	Preclinical	13	Moderate	9	Moderate	28	Low	Not Burnout
222	21	Female	Preclinical	10	Moderate	7	Moderate	22	Moderate	Not Burnout
223	22	Female	Preclinical	0	Low	18	High	18	Moderate	Not Burnout
224	21	Male	Preclinical	12	Moderate	13	High	16	High	Not Burnout
225	22	Female	Preclinical	10	Moderate	12	High	16	High	Not Burnout
226	21	Female	Preclinical	21	High	19	High	1	High	Burnout
227	20	Male	Preclinical	11	Moderate	13	High	11	High	Not Burnout
228	21	Male	Preclinical	19	High	14	High	20	Moderate	Burnout
229	21	Female	Preclinical	28	High	21	High	15	High	Burnout
230	20	Female	Preclinical	20	High	10	Moderate	17	Moderate	Not Burnout
231	19	Female	Preclinical	19	High	14	High	19	Moderate	Burnout

232	21	Female	Preclinical	18	High	12	High	19	Moderate	Burnout
233	22	Male	Preclinical	5	Low	7	Moderate	20	Moderate	Not Burnout
234	21	Female	Preclinical	27	High	17	High	4	High	Burnout
235	20	Female	Preclinical	15	Moderate	11	Moderate	20	Moderate	Not Burnout
236	21	Female	Preclinical	19	High	12	High	22	Moderate	Burnout
237	21	Female	Preclinical	21	High	15	High	15	High	Burnout
238	21	Male	Preclinical	18	High	20	High	20	Moderate	Burnout
239	21	Female	Preclinical	9	Low	9	Moderate	22	Moderate	Not Burnout
240	21	Male	Preclinical	16	Moderate	9	Moderate	16	High	Not Burnout
241	21	Male	Preclinical	10	Moderate	14	High	16	High	Not Burnout
242	21	Female	Preclinical	18	High	8	Moderate	16	High	Not Burnout
243	21	Female	Preclinical	21	High	10	Moderate	24	Low	Not Burnout
244	21	Male	Preclinical	4	Low	17	High	17	Moderate	Not Burnout
245	20	Female	Preclinical	18	High	8	Moderate	18	Moderate	Not Burnout
246	20	Female	Preclinical	19	High	16	High	17	Moderate	Burnout
247	20	Female	Preclinical	15	Moderate	12	High	14	High	Not Burnout
248	20	Female	Preclinical	14	Moderate	15	High	19	Moderate	Not Burnout
249	21	Female	Preclinical	14	Moderate	12	High	18	Moderate	Not Burnout
250	20	Female	Preclinical	20	High	13	High	17	Moderate	Burnout
251	21	Female	Preclinical	6	Low	7	Moderate	26	Low	Not Burnout
252	22	Female	Preclinical	2	Low	0	Low	23	Moderate	Not Burnout
253	20	Female	Preclinical	13	Moderate	8	Moderate	16	High	Not Burnout
254	21	Female	Preclinical	19	High	16	High	26	Low	Burnout
255	21	Female	Preclinical	15	Moderate	13	High	17	Moderate	Not Burnout
256	20	Female	Preclinical	30	High	22	High	7	High	Burnout
257	22	Male	Preclinical	15	Moderate	16	High	23	Moderate	Not Burnout
258	20	Female	Preclinical	13	Moderate	8	Moderate	26	Low	Not Burnout
259	21	Female	Preclinical	12	Moderate	4	Low	17	Moderate	Not Burnout
260	21	Male	Preclinical	4	Low	7	Moderate	32	Low	Not Burnout
261	22	Female	Preclinical	14	Moderate	6	Moderate	28	Low	Not Burnout
262	21	Male	Preclinical	2	Low	11	Moderate	30	Low	Not Burnout
263	20	Female	Preclinical	14	Moderate	5	Moderate	24	Low	Not Burnout
264	21	Male	Preclinical	2	Low	6	Moderate	25	Low	Not Burnout
265	20	Female	Preclinical	18	High	2	Low	21	Moderate	Not Burnout
266	21	Female	Preclinical	14	Moderate	11	Moderate	13	High	Not Burnout
267	21	Female	Preclinical	20	High	20	High	14	High	Burnout
268	22	Male	Preclinical	21	High	23	High	17	Moderate	Burnout
269	21	Female	Preclinical	10	Moderate	3	Low	26	Low	Not Burnout
270	21	Male	Preclinical	24	High	21	High	22	Moderate	Burnout
271	26	Male	Preclinical	17	Moderate	16	High	23	Moderate	Not Burnout
272	22	Female	Preclinical	12	Moderate	8	Moderate	12	High	Not Burnout
273	21	Female	Preclinical	14	Moderate	20	High	25	Low	Not Burnout
274	22	Female	Preclinical	7	Low	1	Low	23	Moderate	Not Burnout
275	21	Male	Clinical	3	Low	0	Low	32	Low	Not Burnout
276	22	Male	Clinical	18	High	9	Moderate	25	Low	Not Burnout
277	21	Male	Clinical	16	Moderate	5	Moderate	20	Moderate	Not Burnout
278	23	Male	Clinical	15	Moderate	8	Moderate	16	High	Not Burnout

279	22	Male	Clinical	15	Moderate	0	Low	25	Low	Not Burnout
280	21	Male	Clinical	1	Low	0	Low	32	Low	Not Burnout
281	21	Male	Clinical	8	Low	2	Low	31	Low	Not Burnout
282	21	Male	Clinical	6	Low	0	Low	27	Low	Not Burnout
283	23	Male	Clinical	13	Moderate	0	Low	12	High	Not Burnout
284	22	Male	Clinical	10	Moderate	2	Low	28	Low	Not Burnout
285	22	Male	Clinical	16	Moderate	2	Low	25	Low	Not Burnout
286	22	Male	Clinical	0	Low	0	Low	18	Moderate	Not Burnout
287	22	Male	Clinical	9	Low	3	Low	25	Low	Not Burnout
288	21	Male	Clinical	5	Low	5	Moderate	30	Low	Not Burnout
289	23	Male	Clinical	17	Moderate	5	Moderate	30	Low	Not Burnout
290	22	Male	Clinical	14	Moderate	4	Low	29	Low	Not Burnout
291	21	Male	Clinical	7	Low	7	Moderate	11	High	Not Burnout
292	23	Male	Clinical	12	Moderate	0	Low	29	Low	Not Burnout
293	22	Male	Clinical	13	Moderate	1	Low	21	Moderate	Not Burnout
294	22	Male	Clinical	25	High	0	Low	29	Low	Not Burnout
295	21	Male	Clinical	2	Low	0	Low	24	Low	Not Burnout
296	21	Male	Clinical	8	Low	4	Low	32	Low	Not Burnout
297	22	Male	Clinical	2	Low	3	Low	16	High	Not Burnout
298	22	Male	Clinical	24	High	10	Moderate	23	Moderate	Not Burnout
299	23	Male	Clinical	6	Low	0	Low	33	Low	Not Burnout
300	21	Male	Clinical	21	High	6	Moderate	28	Low	Not Burnout
301	22	Female	Clinical	15	Moderate	6	Moderate	21	Moderate	Not Burnout
302	22	Female	Clinical	14	Moderate	4	Low	22	Moderate	Not Burnout
303	22	Female	Clinical	20	High	16	High	24	Low	Burnout
304	22	Female	Clinical	3	Low	1	Low	8	High	Not Burnout
305	22	Female	Clinical	6	Low	4	Low	11	High	Not Burnout
306	21	Female	Clinical	5	Low	0	Low	25	Low	Not Burnout
307	24	Female	Clinical	17	Moderate	8	Moderate	17	Moderate	Not Burnout
308	23	Female	Clinical	5	Low	5	Moderate	26	Low	Not Burnout
309	22	Female	Clinical	11	Moderate	4	Low	24	Low	Not Burnout
310	22	Female	Clinical	11	Moderate	0	Low	31	Low	Not Burnout
311	23	Female	Clinical	20	High	13	High	26	Low	Burnout
312	22	Female	Clinical	22	High	8	Moderate	18	Moderate	Not Burnout
313	23	Female	Clinical	18	High	7	Moderate	25	Low	Not Burnout
314	23	Female	Clinical	19	High	0	Low	24	Low	Not Burnout
315	22	Female	Clinical	19	High	1	Low	22	Moderate	Not Burnout
316	25	Female	Clinical	6	Low	0	Low	22	Moderate	Not Burnout
317	22	Female	Clinical	23	High	14	High	17	Moderate	Burnout
318	22	Female	Clinical	16	Moderate	0	Low	21	Moderate	Not Burnout
319	23	Female	Clinical	11	Moderate	0	Low	36	Low	Not Burnout
320	23	Female	Clinical	22	High	0	Low	21	Moderate	Not Burnout
321	22	Female	Clinical	3	Low	0	Low	15	High	Not Burnout
322	22	Female	Clinical	10	Moderate	0	Low	24	Low	Not Burnout
323	22	Female	Clinical	25	High	5	Moderate	22	Moderate	Not Burnout
324	21	Female	Clinical	11	Moderate	1	Low	22	Moderate	Not Burnout
325	22	Female	Clinical	18	High	9	Moderate	21	Moderate	Not Burnout

326	22	Female	Clinical	13	Moderate	4	Low	26	Low	Not Burnout
327	22	Female	Clinical	15	Moderate	9	Moderate	19	Moderate	Not Burnout
328	22	Female	Clinical	24	High	15	High	29	Low	Burnout
329	22	Female	Clinical	1	Low	0	Low	11	High	Not Burnout
330	22	Female	Clinical	12	Moderate	0	Low	30	Low	Not Burnout
331	20	Female	Clinical	18	High	0	Low	31	Low	Not Burnout
332	21	Female	Clinical	12	Moderate	4	Low	21	Moderate	Not Burnout
333	22	Female	Clinical	19	High	12	High	24	Low	Burnout
334	22	Female	Clinical	11	Moderate	2	Low	31	Low	Not Burnout
335	22	Female	Clinical	15	Moderate	2	Low	23	Moderate	Not Burnout
336	24	Female	Clinical	11	Moderate	4	Low	21	Moderate	Not Burnout
337	22	Female	Clinical	6	Low	0	Low	34	Low	Not Burnout
338	22	Female	Clinical	12	Moderate	0	Low	27	Low	Not Burnout
339	21	Female	Clinical	14	Moderate	7	Moderate	23	Moderate	Not Burnout
340	21	Female	Clinical	19	High	3	Low	24	Low	Not Burnout
341	22	Female	Clinical	14	Moderate	8	Moderate	12	High	Not Burnout
342	22	Female	Clinical	4	Low	0	Low	22	Moderate	Not Burnout
343	21	Female	Clinical	9	Low	4	Low	22	Moderate	Not Burnout
344	22	Female	Clinical	17	Moderate	9	Moderate	32	Low	Not Burnout
345	21	Female	Clinical	16	Moderate	6	Moderate	22	Moderate	Not Burnout
346	22	Female	Clinical	30	High	11	Moderate	21	Moderate	Not Burnout
347	22	Female	Clinical	23	High	16	High	32	Low	Burnout
348	23	Female	Clinical	11	Moderate	5	Moderate	19	Moderate	Not Burnout
349	21	Female	Clinical	12	Moderate	5	Moderate	35	Low	Not Burnout
350	22	Female	Clinical	14	Moderate	0	Low	14	High	Not Burnout
351	21	Female	Clinical	10	Moderate	0	Low	32	Low	Not Burnout
352	23	Female	Clinical	21	High	12	High	14	High	Burnout
353	23	Female	Clinical	5	Low	0	Low	25	Low	Not Burnout
354	22	Female	Clinical	20	High	1	Low	10	High	Not Burnout
355	21	Female	Clinical	8	Low	2	Low	23	Moderate	Not Burnout
356	21	Female	Clinical	8	Low	6	Moderate	12	High	Not Burnout
357	21	Female	Clinical	9	Low	0	Low	27	Low	Not Burnout
358	22	Female	Clinical	13	Moderate	2	Low	21	Moderate	Not Burnout
359	24	Male	Clinical	11	Moderate	6	Moderate	13	High	Not Burnout
360	23	Male	Clinical	14	Moderate	3	Low	20	Moderate	Not Burnout
361	23	Male	Clinical	13	Moderate	5	Moderate	15	High	Not Burnout
362	23	Male	Clinical	15	Moderate	2	Low	17	Moderate	Not Burnout
363	23	Male	Clinical	12	Moderate	3	Low	20	Moderate	Not Burnout
364	22	Male	Clinical	12	Moderate	4	Low	30	Low	Not Burnout
365	23	Male	Clinical	9	Low	0	Low	21	Moderate	Not Burnout
366	23	Male	Clinical	19	High	2	Low	26	Low	Not Burnout
367	23	Male	Clinical	14	Moderate	7	Moderate	17	Moderate	Not Burnout
368	23	Male	Clinical	0	Low	0	Low	36	Low	Not Burnout
369	23	Male	Clinical	13	Moderate	0	Low	9	High	Not Burnout
370	25	Male	Clinical	4	Low	0	Low	29	Low	Not Burnout
371	23	Male	Clinical	14	Moderate	6	Moderate	25	Low	Not Burnout
372	23	Male	Clinical	10	Moderate	3	Low	32	Low	Not Burnout

373	23	Female	Clinical	7	Low	0	Low	33	Low	Not Burnout
374	23	Female	Clinical	19	High	9	Moderate	26	Low	Not Burnout
375	23	Female	Clinical	17	Moderate	3	Low	22	Moderate	Not Burnout
376	23	Female	Clinical	13	Moderate	4	Low	10	High	Not Burnout
377	23	Female	Clinical	26	High	10	Moderate	19	Moderate	Not Burnout
378	22	Female	Clinical	13	Moderate	2	Low	25	Low	Not Burnout
379	20	Female	Clinical	4	Low	0	Low	12	High	Not Burnout
380	22	Female	Clinical	6	Low	0	Low	35	Low	Not Burnout
381	23	Female	Clinical	12	Moderate	2	Low	18	Moderate	Not Burnout
382	22	Female	Clinical	14	Moderate	2	Low	26	Low	Not Burnout
383	23	Female	Clinical	17	Moderate	4	Low	20	Moderate	Not Burnout
384	23	Female	Clinical	18	High	1	Low	25	Low	Not Burnout
385	24	Female	Clinical	5	Low	0	Low	17	Moderate	Not Burnout
386	24	Female	Clinical	13	Moderate	15	High	21	Moderate	Not Burnout
387	23	Female	Clinical	10	Moderate	3	Low	27	Low	Not Burnout
388	29	Female	Clinical	18	High	0	Low	15	High	Not Burnout
389	22	Female	Clinical	15	Moderate	12	High	24	Low	Not Burnout
390	23	Female	Clinical	17	Moderate	4	Low	18	Moderate	Not Burnout
391	24	Female	Clinical	15	Moderate	9	Moderate	14	High	Not Burnout
392	23	Female	Clinical	11	Moderate	4	Low	22	Moderate	Not Burnout
393	23	Female	Clinical	11	Moderate	9	Moderate	21	Moderate	Not Burnout
394	22	Female	Clinical	26	High	15	High	16	High	Burnout
395	23	Female	Clinical	25	High	1	Low	19	Moderate	Not Burnout
396	23	Female	Clinical	2	Low	0	Low	30	Low	Not Burnout
397	23	Female	Clinical	7	Low	0	Low	19	Moderate	Not Burnout
398	23	Female	Clinical	9	Low	0	Low	22	Moderate	Not Burnout
399	22	Female	Clinical	11	Moderate	6	Moderate	23	Moderate	Not Burnout
400	24	Female	Clinical	20	High	10	Moderate	16	High	Not Burnout
401	24	Female	Clinical	4	Low	1	Low	25	Low	Not Burnout
402	22	Female	Clinical	8	Low	1	Low	24	Low	Not Burnout
403	23	Female	Clinical	2	Low	1	Low	22	Moderate	Not Burnout
404	23	Female	Clinical	16	Moderate	4	Low	25	Low	Not Burnout
405	23	Female	Clinical	10	Moderate	0	Low	16	High	Not Burnout
406	23	Female	Clinical	10	Moderate	16	High	18	Moderate	Not Burnout
407	24	Female	Clinical	16	Moderate	8	Moderate	17	Moderate	Not Burnout
408	23	Female	Clinical	11	Moderate	0	Low	22	Moderate	Not Burnout
409	22	Female	Clinical	16	Moderate	11	Moderate	17	Moderate	Not Burnout
410	23	Female	Clinical	16	Moderate	10	Moderate	16	High	Not Burnout
411	23	Female	Clinical	14	Moderate	4	Low	12	High	Not Burnout
412	23	Female	Clinical	6	Low	1	Low	26	Low	Not Burnout
413	23	Female	Clinical	7	Low	3	Low	29	Low	Not Burnout
